# Spectrum Handoff based on Imperfect Channel State Prediction Probabilities with Collision Reduction in Cognitive Radio Ad Hoc Networks

**DOI:** 10.3390/s19214741

**Published:** 2019-10-31

**Authors:** Atif Shakeel, Riaz Hussain, Adeel Iqbal, Irfan Latif Khan, Qadeer Ul Hasan, Shahzad Ali Malik

**Affiliations:** Department of Electrical and Computer Engineering, COMSATS University Islamabad (CUI), Islamabad 45550, Pakistan; rhussain@comsats.edu.pk (R.H.); adeeliqbal@comsats.edu.pk (A.I.); irfan_latif@comsats.edu.pk (I.L.K.); qadeer.hasan@comsats.edu.pk (Q.U.H.); smalik@comsats.edu.pk (S.A.M.)

**Keywords:** ad hoc networks, cognitive radio, channel state prediction, channel access, extended data delivery time, spectrum handoff, spectrum management

## Abstract

The spectrum handoff is highly critical as well as challenging in a cognitive radio ad hoc network (CRAHN) due to lack of coordination among secondary users (SUs), which leads to collisions among the SUs and consequently affects the performance of the network in terms of spectrum utilization and throughput. The target channel selection mechanism as part of handoff process can play an enormously significant role in minimizing the collisions among the SUs and improving the performance of a cognitive radio network (CRN). In this paper, an enhanced target channel selection scheme based on imperfect channel state prediction is proposed for the spectrum handoff among the SUs in a CRAHN. The proposed scheme includes an improved frame structure that increases coordination among the SUs in the ad hoc environment and helps in organizing the SUs according to the shortest job first principle during channel access. Unlike the existing prediction-based spectrum handoff techniques, the proposed scheme takes into account the accuracy of channel state prediction; the SUs affected due to false prediction are compensated by allowing them to contend for channel access within the same transmission cycle and thus enabling them to achieve higher throughput. The proposed scheme has been compared with the contemporary spectrum handoff schemes and the results have demonstrated substantial improvement in throughput and extended data delivery time by virtue of the reduced number of collisions.

## 1. Introduction

Over the last few decades, the demand for wireless spectrum access has grown exponentially. This rise in demand is due to enormous increase in number of end users from healthcare, businesses, financial, defense, internet of things (IoT), etc., having seamless wireless connectivity requirements, running different interactive applications [1]. However, the fixed spectrum allocation policies, which aimed to prevent interference with other users, resulted in spectrum scarcity [2]. Spectrum allocated to primary users (PUs) is mostly underutilized in time and space. According to federal communication commission (FCC), there is a huge variation in the temporal and spatial spectrum utilization, ranging from 15% to 85% [3,4]. This forced the authorities to look for better spectrum management policies.

Cognitive radio (CR) is a key technology that realizes the concept of dynamic spectrum access (DSA), in which secondary users (SUs) can access the underutilized portion of the spectrum of a primary network through sensing and channel access mechanisms [5,6]. Quick and accurate spectrum sensing is extremely important in a cognitive radio network (CRN) sensing phase to maximize the pool of resources available to SUs [7,8]. In a CRN, the SUs opportunistically access channels that are sensed to be currently unutilized by the primary users (PUs); however, PUs can preempt the SUs’ transmissions any time and, consequently, the SUs have to vacate the channels to avoid interference to the PUs and switch to new idle channels to resume their transmissions. This process of switching to a new idle channel by the SU is called spectrum handoff, which is the primary focus of this work.

Spectrum handoff is mainly classified into two main categories: (i) non-channel switching, also known as non-handoff (NHO); and (ii) channel switching spectrum handoff [9]. In NHO, the interrupted SU stays on the same channel and waits for the PU to vacate that channel to resume its unfinished transmission. In this case, the current channel and the target channel is the same. However, in channel switching spectrum handoff the interrupted SU has to vacate the channel and switch to a new idle channel. The process of finding the new idle channel can be either reactive or proactive [10]. In reactive channel switching spectrum handoff, the search for the new idle channel is done in real time on the actual arrival of PU. It provides an accurate list of idle channels as the spectrum sensing is performed in the most relevant environment. However, it increases the handoff delay, which in turn increases the extended data delivery time (EDDT). In the proactive channel switching spectrum handoff, the SU predicts the channel state based on the long-term PU traffic statistics and target channel is selected well in advance. Therefore, when the SU is interrupted, it switches to one of the already selected target channels. This saves spectrum sensing time that incurred in the case of reactive channel switching spectrum handoff. Here, the trade-off lies in prediction accuracy.

Prediction accuracy is a function of PU traffic intensity and greatly affects the performance of the network, especially during spectrum handoff. Existing work on spectrum handoff where channel state prediction is used for proactive target channel selection ignores the accuracy of prediction, considering only a perfect prediction mechanism [11,12,13,14,15]. Imperfect channel prediction mechanism has been considered for improving only the spectrum sensing phase in a cognitive cycle [16,17,18,19]. In [20,21], prediction accuracy for improving the spectrum sensing delay as a part of spectrum handoff has been considered; however, this is applicable only in a centralized CRN. The work in this paper differs from the existing work in that the imperfect channel prediction method is being considered for analyzing the spectrum handoff in a multi-user cognitive radio ad hoc network (CRAHN). To the best of our knowledge, this is the first work, which takes into consideration the channel state prediction accuracy and the network coordination among distributed SUs leading to the improvement of collision probability, EDDT and throughput of the system. The coordination among distributed SUs during channel selection and access is achieved by proposing a novel frame structure which provides contention free channel access when the channel state prediction is true; otherwise, it offers a contention-based channel access within the same transmission cycle. Furthermore, the impact of PU traffic intensity on the performance of a CRAHN has also been considered in this work.

### Contributions

The following are the major contributions of this research:

- A spectrum handoff scheme based on imperfect channel state prediction is proposed, which aims to reduce the EDDT and improve the average throughput of the system by virtue of the reduced number of collisions among SUs.
- An improved frame structure is proposed that aims at providing coordination among distributed SUs and organizing them according to shortest job first (SJF) principle during channel access.
- The performance of the proposed spectrum handoff scheme was evaluated through modeling and simulation; a comparison with the existing schemes was also carried out that demonstrates improvement in EDDT, the number of collisions among SUs and average throughput of the SUs in a CRAHN.

The rest of the paper is organized as follows. The related work is presented in Section 2 with comparative analysis of existing spectrum handoff techniques. The proposed spectrum handoff scheme, the system model and assumptions are described in detail in Section 3. The results are presented in Section 4, followed by conclusion and future direction in Section 5.

## 2. Related Work

Existing work shows that many efforts have been made in recent years to reduce the number of collisions, lower extended data delivery time and improve the overall throughput of the system during spectrum handoff management process for both centralized and decentralized CRNs. In centralized CRN, a central entity is coordinating the channel selection and access process and helps in reducing unwanted collisions among SUs. However, in a decentralized CRN, also known as CRAHN, due to the distributed nature of the network, SUs have to bear the burden of fair channel selection and access mechanism. Network coordination among distributed SUs is a very challenging task and is achieved using split phase, common hopping sequence and dedicated common control channel (CCC) [22,23]. Split phase and common hopping sequence require tight network synchronization for all the network nodes. The dedicated channel is favorable and outperforms the other two when there are many primary channels; otherwise, it limits the spectral efficiency when number of channels is small [24]. In [25,26], the authors considered common hopping mechanism to find channel rendezvous during spectrum handoff management. In [27,28], a dedicated CCC is considered for coordination among distributed SUs. [29] concluded that, with dedicated CCC, network coordination can be performed simultaneously with data transmission, which enables SUs to achieve higher throughput, thus making use of a dedicated CCC for network coordination in a CRAHN is quite favorable.

Target channel selection process as a part of spectrum handoff can be performed reactively or proactively. Reactive target channel selection gives accurate state of the channel due to real-time sensing [30,31,32], whereas proactive channel selection depends on accuracy of channel state prediction that plays important role in overall performance of spectrum handoff [11,12,13,14,15]. Due to fluctuating nature of radio environment and PU activities, the impact of false prediction cannot be ignored. In [30], an analytical model is presented to characterize the affect of multiple interruptions caused by the PUs on the extended data delivery time by taking into consideration the reactive decision spectrum handoff. Coordination among SUs is not considered in this work. The authors of [31] presented an analytical framework to evaluate the effect of reactive spectrum handoff on real-time traffic in a CRN. The reactive sensing during target channel selection reduces the blocking and forced termination probabilities and improves the channel utilization. The authors of [32] proposed a reactive handoff scheme using dedicated CCC, where SUs can hold multiple channels simultaneously even in the presence of PU using hybrid sharing scheme, which increases the net throughput of the system and keeps the interference temperature within acceptable limits. The authors of [11,12] proposed a proactive spectrum handoff in which target channel selection is achieved proactively, which saves sensing and handshake time during spectrum handoff. The impact of false prediction during target channel selection process, which causes collision among PU and SUs, is ignored in this work. Similarly, the authors of [13] proposed a channel state prediction based spectrum handoff technique for CRAHN, where SUs organize themselves through pseudo-random sequence during channel access. The knowledge of such sequence must be known to each SU prior to channel access in each time slot, which can be challenging in a distributed network. The proposed scheme focuses on minimizing the frequent spectrum handoff by selecting the channel with maximum residual time. This scheme works better when compared with existing reactive spectrum handoff schemes in terms of average throughput and service time. Accuracy of channel state prediction is not considered in this work as well. In [25], the authors presented a prediction based proactive spectrum handoff for distributed secondary network. A distributed channel selection mechanism is presented to avoid collisions among SUs. Common hopping is used to find channel rendezvous. SUs affected due to prediction error attempt for channel access in the next time cycle. EDDT and number of collisions have been reduced compared to reactive spectrum handoff. The authors of [14] proposed an adaptive spectrum handoff strategy that combines the benefits of both reactive and proactive channel switching. The authors used primary prioritized Markov approach to analyze the interaction between PUs and SUs. The accuracy of channel state prediction for proactive channel switching is not considered in this work either. In [15], a spectrum handoff technique is proposed by combining the advantages of reactive and proactive target channel selection process. This scheme considers perfect channel state prediction mechanism for finding the list of idle channels. The prediction accuracy, which directly impacts the performance of network, is not considered. The authors of [11,12,13,14,15] did not consider the impact of false channel state prediction on the performance. Most of the studies based on channel state prediction have limitations due to possibility of false prediction, as studied in [33]. It is very challenging to get an accurate result of spectrum prediction due to time varying nature of radio environment. In addition, knowledge of perfect channel state information of primary network is difficult as the two networks work independently [34].

In [16], the authors proposed a frame structure to exploit the cooperative spectrum prediction and sensing mechanism for a centralized CRN, where the secondary base station predicts the channel state. Cooperative sensing is used to improve the prediction accuracy by reducing the sensing errors. In this work, the imperfect channel state prediction using artificial neural network (ANN) is considered and used for hybrid spectrum sharing mechanism. However, spectrum handoff process is not elaborated. In [17], the authors proposed a channel state predictor using ANN and Hidden Markov model (HMM) and investigated the accuracy of both models and evaluated their performances. The advantages of channel state prediction in this work is applied only to the spectrum sensing process by improving the sensing time. Yang et al. [18] proposed a frame structure that incorporates prediction phase to select channels for real time sensing instead of sensing all the channels, thus reducing spectrum sensing time. Similarly, [19] considered HMM for channel state prediction and its impact on the improvement of sensing delay by skipping the channels predicted busy in the sensing phase, thus only sensing the channels predicted idle. The authors of [16,17,18,19] considered the impact of prediction accuracy; however, benefits of prediction is limited to improve the spectrum sensing time, ignoring the spectrum handoff management process. The authors of [20] proposed a proactive channel switching handoff mechanism to minimize the number of handoff. A list of candidate target channel based on probability of idleness is maintained and sensed during handoff instead of sensing all channels. This reduces the sensing delay as a part of handoff process. However, this work considers centralized network architecture as well. In [21], a probability based proactive spectrum handoff mechanism is proposed where a centralized device computes the probability of idleness for each primary channel and then based on QoS requirements of SUs allocates appropriate predicted channel during handoff. Handoff delay and transmission delay are improved by sensing the right channel for handoff based on prediction probabilities. However, this scheme is targeted for a centralized CRN.

In a centralized CRN, a central entity provides coordination among SUs during random channel access, which helps to avoid collisions among the SUs and maximizes the utilization of discovered idle channels. In an ad hoc CRN with no central entity, coordination among SUs during channel access is a challenging task that must be handled with caution to prevent collisions among the SUs. Avoiding collisions becomes even more critical during spectrum handoff than that in general channel allocation scenarios as these collisions result in loss of created opportunities and increase the EDDT for SU packets. The probability of collision during random channel access for $N_{su}$ SUs contending for *M* sensed idle channel as in [35,36] is given by:

(1) $$P_{cx}=1-\left[{\left(\frac{M-1}{M}\right)}^{N_{su}}+{\frac{\left(\frac{M-1}{M}\right)}{M-1}}^{N_{su}}\right].$$

It is observed that the probability of collision increases as the number of contenders increases for a fixed number of idle channels. This in turn decreases the average number of successful SUs. The number of idle channels available to SUs in a CRN depends on the primary user traffic intensity. At high primary load, there are very few idle channels, hence the probability of collision increases. The work in this paper is focused on developing a spectrum handoff scheme that minimizes the amount of collisions among the SUs during contention for channel access. If some of the SUs are provided with contention free channel access on predicted idle channels, leaving behind fewer contenders for contention based channel access, thus leading to better spectrum handoff performance.

Equation (1) can be rewritten as:

(2) $$P_{cx_{new}}=1-\left[{\left(\frac{M_{n}-1}{M_{n}}\right)}^{N_{su}-N_{cf}}+{\frac{\left(\frac{M_{n}-1}{M_{n}}\right)}{M_{n}-1}}^{N_{su}-N_{cf}}\right],$$

where $M_{n}=M-N_{cf}$ are the remaining available idle channels, $N_{cf}$ is the number of SUs getting a contention free channel access and $P_{cx_{new}}$ is the new probability of collision having fewer contenders compared to Equation (1). Based on this philosophy, we have devised a spectrum handoff scheme with due consideration of imperfect channel state prediction. It provides contention free channel access to fraction of SUs, where channel state prediction is true. SUs not getting contention free access during handoff due to false prediction contend for the sensed idle channel in random fashion within the same transmission cycle. This improves the overall spectrum utilization and translates into reduced data delivery time for the SUs in a CRAHN.

A comparison of the the proposed scheme with related work in terms of various aspects is presented in Table 1. The related work has been categorized based on the main idea of research, use of imperfect spectrum prediction, benefits of prediction for spectrum handoff, channel access re-attempt within same time cycle, improved frame structure for ad hoc network, network architecture, target channel selection mechanism, performance evaluation of EDDT, throughput, percentage of collisions among SUs, cycle time utilization efficiency. Our work encompasses all of these aspects.

## 3. Proposed Scheme

In this section, we present our proposed proactive spectrum handoff scheme which considers an imperfect channel state prediction and aims to reduce the collisions among SUs during spectrum handoff. This translates into improved EDDT and higher average throughput of the CRAHN. The network model, assumptions, improved frame structure, channel state prediction and target channel selection mechanism are presented below.

### 3.1. Network Model and Assumptions

**Figure 1:**
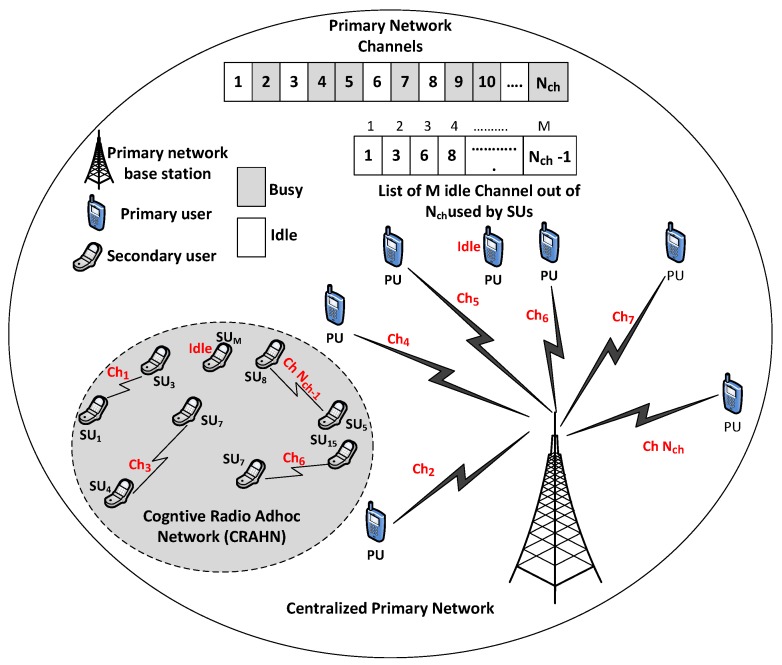
Network model.

We consider a centralized primary network with $N_{ch}$ licensed channels for the PUs, a distributed secondary network having *M* idle channels, discovered out of $N_{ch}$ licensed channels in a transmission cycle $\left(T_{cycle}\right)$, available to SUs, as depicted in Figure 1. To characterize the effect of multiple interruptions caused by PUs to SUs, we consider slot-based modeling technique [12], where presence of PU at any channel is only checked at the beginning of each slot known as $T_{cycle}$. Due to the decentralized nature of the secondary network, coordination among the SUs is achieved through a dedicated global CCC, which is leased from the primary network and assumed to be available to all SUs [29]. To simplify the analysis, we have assumed spectrum sensing process to be perfect by ignoring the sensing errors, i.e., miss detection and false alarms [37]. Further PU traffic is considered as a binary stochastic process, i.e., being either idle ($H_{0}$) or busy ($H_{1}$) [38]. In addition, PU’s arrival process is modeled using Poisson distribution with parameter ($\lambda_{p}$) and channel holding time, which is the expected duration of a PU present on the channel, is modeled as exponential distribution with parameter ($\mu_{p}$) [39].

**Figure 2:**
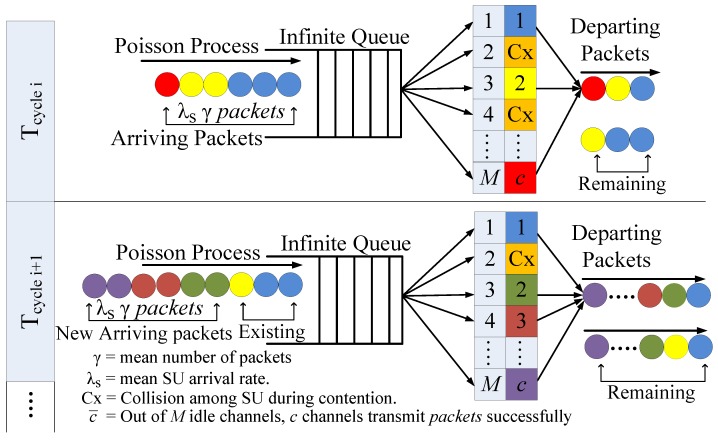
*PRI-M/M/c* queuing model of secondary network.

The probability of channel being in idle state is $P\left(H_{0}\right)=\left(\lambda_{p}-\mu_{p}\right)/\lambda_{p}$ and being in busy state is $P\left(H_{1}\right)=\mu_{p}/\lambda_{p}$. For a secondary network, the preemptive resume identical (PRI)M/M/c queuing model is considered, as shown in Figure 2. SUs can be preempted by PUs during their transmission; therefore, they have to either wait on the current channel to become idle again or switch to target channel to resume the unfinished transmission. For service policy, each channel has two queues: high priority queue for PUs and low priority queue for SUs. However, the service policy within the same priority queue is assumed to be first come first served (FCFS).

The average number of packets to be transmitted by an SU is denoted by γ, and the SU arrival rate in each $T_{cycle}$ is a Poisson process with parameter $\lambda_{s}$. Thus, the number of packets entering into the system during a $T_{cycle}$ is the product of $\lambda_{s}$ and γ. The number of idle channels discovered (M) is a function of load on primary network (ρ) and varies in $T_{cycle}$.

### 3.2. Proposed Frame Format

**Figure 3:**
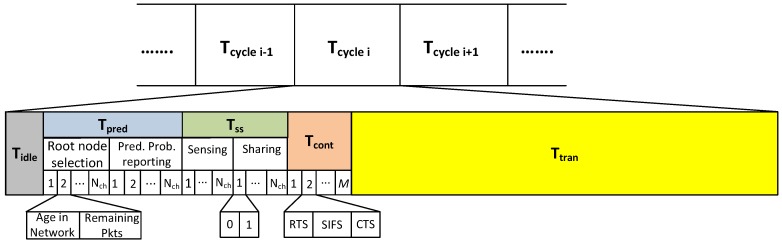
Proposed frame format.

The frame format of proposed spectrum handoff scheme is shown in Figure 3, which has been built upon the frame structure presented in Figure 1 of [18]. The proposed frame format consists of five phases: $T_{idle}$, $T_{pred}$, $T_{ss}$, $T_{cont}$ and $T_{tran}$. $T_{idle}$ and $T_{cont}$ phases have been added in such a way that it can work in ad hoc network environment as well as in the case of prediction error to provide a second chance to unsuccessful SUs during contention within the same $T_{cycle}$.

#### 3.2.1. $T_{idle}$

Each $T_{cycle}$ begins with an idle phase $T_{idle}$, which is used by SUs to synchronize they before start sending control information. The length of this phase is:

(3) $$T_{idle}=aSIFSTime+2*aSlotTime,$$

where aSIFSTime and aSlotTime are the short inter-frame space time and the slot time, respectively, as in [40].

#### 3.2.2. $T_{pred}$

Prediction phase ($T_{pred}$) is divided in to two sub-phases: (i) root node selection; and (ii) channel state prediction probabilities reporting phase. Considering the distributed nature of the network, one of the SUs has to be elected as a root node. The responsibility of root node is to predict the probability of idleness of primary channels and share this information among other SUs in the system. The probability of idleness, it is calculated based on the sensing information of the previous $T_{cycle}$. Therefore, an SU which is present in the system for more $T_{cycle}$ is the best candidate for predicting the channel state probabilities. The field “age in network” is used to elect the root node. The oldest node is the best choice for root node as it has the channel state information for more number of $T_{cycle}$, which in turn leads to better prediction. Due to the decentralized nature of the ad hoc network, there must be some node providing coordination among SUs during random channel access to avoid collisions. As there is no central coordinator in an ad hoc network, a node has to be elected for reporting the predicted probabilities to all SUs, thus a root node is selected. For this purpose, SUs which are already in the system and were also successful in channel access in the previous $T_{cycle}$ share the information about their age in the network and the number of remaining packets to be transmitted in the sub-slot corresponding to the channel number used by SU in the last $T_{cycle}$. Note that this sub phase has $N_{ch}$ sub-slots. As the oldest SU in the system is the best candidate for prediction, the SU with highest value of age in the network is selected as a root node.

In the case of contention, where two or more SU have identical age, the dispute is resolved with the priority given to the one using the smallest channel number. The number of remaining packets field is used to organize SUs according to shortest job first.

In the channel prediction probabilities reporting phase, the root node predicts the probability of idleness for each channel $\left(1\leq channel\leq N_{ch}\right)$ based on the channel usage history, which is known to the root node through channel sensing data collected in the previous $nT_{cycles}$. Root node shares the predicted probability of idleness of each channel in the corresponding sub-slot of the second sub-phase. At the end of this phase, SU selects the target channel according to Algorithm 1. Unlike existing proactive handoff, where prediction results are assumed perfect, our scheme considers the prediction error. The length of this phase $T_{pred}$ is as follows:

(4) $$T_{pred}=N_{ch}*15*aBitsTime,$$

where aBitsTime is the time to transmit one bit for a given data rate (R) of the network. Each sub-slot of root node selection sub phase is 8 bits as we have assigned 4 bits for the age in network field and 4 bits for the remaining packets field. The sub-slot of prediction probability reporting phase is 7 bits considering a two decimal place precision for the probability value.

#### 3.2.3. $T_{ss}$

$T_{ss}$ is the sensing and sharing phase, which has two sub-phases: (i) sensing; and (ii) sharing. In the first sub-phase, SUs cooperatively sense the spectrum to discover idle channels. Sensing result is shared among users in the sharing sub-phase. If a channel predicted idle is also discovered idle during this phase, it is considered as a true prediction, otherwise a false prediction. This information is shared among other users in the sharing sub-phase. The length of this phase as in [36] is as follows:

(5) $$T_{ss}=N_{ch}*3*aSlotTime.$$

#### 3.2.4. $T_{cont}$

While the successful SUs, where channel state predictions are true, begin their data transmissions right away on their assigned channels, the unsuccessful SUs, which were deprived of channel access due to false prediction, along with the new arriving SUs, contend for channel access in a random fashion in the contention phase $\left(T_{cont}\right)$ [35]. Each contending SU selects a channel from the list of $\left(M-N_{cf}\right)$ idle channels and exchanges RTS and CTS handshake messages and, if successful, transmits its data in $T_{tran}$ on the channel it won during contention. The length of contention phase is long enough to exchange RTS and CTS frames and according to [36] is given by:

(6) $$T_{cont}=M-N_{cf}*\left(RTS+SIFS+CTS\right),$$

where $\left(M-N_{cf}\right)$ is the number of idle channels available for contention phase and RTS and CTS denote the time to send request to send (RTS) and clear to send (CTS) messages.

It is to be noted that a primary feature of the proposed spectrum handoff scheme is that some of the SUs, requiring spectrum handoff, get contention-free access and those unsuccessful in contention-free access get a second chance of channel access in the same $T_{cycle}$, thus significantly reducing the EDDT.

#### 3.2.5. $T_{tran}$

After the successful channel access, either by the virtue of true channel state prediction or by contending in random access fashion, the remaining time in the $T_{cycle}$ is used by the SUs for data transmission. The length of this phase is:

(7) $$T_{tran}=T_{cycle}-T_{overhead},$$

where $T_{overhead}$ is the time for transmitting control information, which depends on handoff scheme. In the proposed and two existing spectrum handoff schemes, i.e., non-handoff (NHO) and random handoff (RHO), which we used for comparative analysis, $T_{overhead}$ is as follows:

(8) $$T_{overhead}^{RHO}=T_{idle}+T_{ss}+T_{cont},$$

(9) $$T_{overhead}^{NHO}=T_{idle}+T_{ss},$$

(10) $$T_{overhead}^{proposed}=T_{pred}+T_{idle}+T_{ss}+T_{cont}.$$

In NHO, SUs follow always-staying strategy for target channel selection, whereas always-changing strategy is followed in RHO scheme, in which target channel is randomly selected. The proposed scheme follows prediction based target channel selection for handoff.

### 3.3. Channel State Prediction

A binary series method [41] for predicting the probability of idleness of a channel is used in the model. In this method, the channel states for last *n*$T_{cycle}$ are analyzed to find the probability of idleness for the channel in $T_{cycle}^{n+1}$ is as follows:

(11) $$S_{n+1}\leftarrow S_{n},S_{n-1},S_{n-2}\ldots \ldots ,S_{2},S_{1}.$$

As per conventional theory of probability [41,42], the probability of channel being idle in the $T_{cycle}^{n+1}$ is defined as a ratio of the number of idle channels discovered to the total number of channels sensed prior to ${\left(n+1\right)}^{th}$ $T_{cycle}$.

(12) $$P_{idleness}^{S_{n+1}}=\sum_{i=1}^{n}\left(\frac{S_{i}}{n}\right),$$

where $S_{i}$ is the state of channel during $T_{cycle}^{i}$, which is “1” if channel is idle and “0” if busy. $P_{Th}$ is the probability threshold. The channel is considered as predicted idle if the $P_{idleness}^{S_{n+1}}$ as given in Equation (12) is greater than $P_{Th}$, otherwise predicted busy. To consider imperfect spectrum prediction, the probability of prediction error $\left(P_{p}^{e}\right)$ is given as:

(13) $$P_{p}^{e}=\left(P_{idleness}^{S_{n+1}}>P_{Th}\right)P\left(H_{1}\right)+\left(P_{idleness}^{S_{n+1}}<P_{Th}\right)P\left(H_{0}\right),$$

where $P(H_{0})$ and $P(H_{1})$ are dependent on the load on primary network (ρ), e.g., at ρ=0.6 on average 60% of the channel are occupied by the PUs and 40% are available to the SUs, i.e., $P(H_{1})=0.6$, similarly $P(H_{0})=0.4$. The probability distribution of true channel state and predicted state is presented in Table 2 [18].

It yields that the probability of channel predicted idle $\left(P_{idleness}^{S_{n+1}}>P_{Th}\right)$ is given as:

(14) $$P_{pred}^{idle}=P\left(H_{0}\right)\left(1-P_{p}^{e}\right)+P\left(H_{1}\right)\left(P_{p}^{e}\right),$$

and the probability of channel predicted as busy $\left(P_{idleness}^{S_{n+1}}<P_{Th}\right)$ is as follows:

(15) $$P_{pred}^{busy}=P\left(H_{0}\right)\left(P_{p}^{e}\right)+P\left(H_{1}\right)\left(1-P_{p}^{e}\right).$$

### 3.4. Target Channel Selection

**Figure 4:**
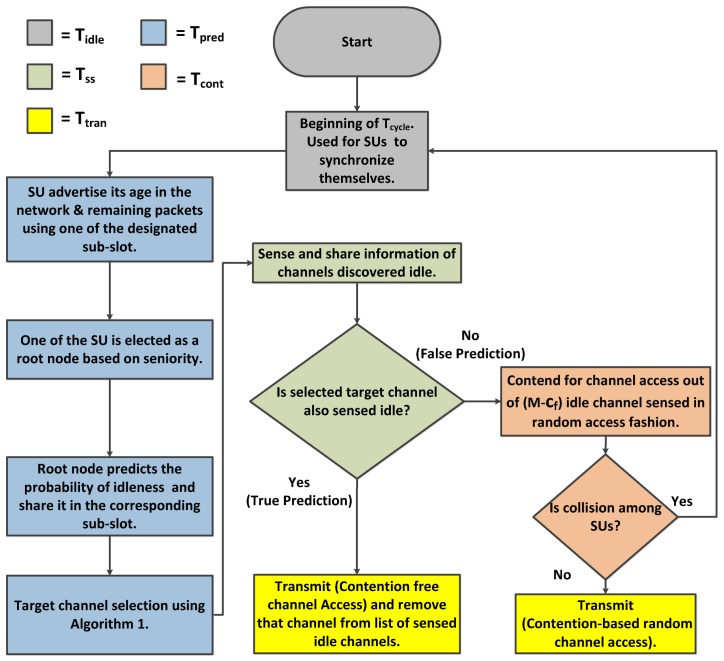
Flow diagram of the proposed model.

Target channel selection is achieved using Algorithm 1, where selected target channel is dependent on the prediction result. In the case of true prediction, contention free channel access will be granted using the shortest job first principle. Channels are arranged in highest to lowest probability of being idle, whereas SUs are arranged from lowest to highest value of the number of remaining packets, i.e., shortest job first. Due to prediction errors, unsuccessful SUs contend for channel access in the same $T_{cycle}$ using procedure FalsePrediction of Algorithm 1, where SUs contend for channel access in random fashion resulting in either success or failure. In the case of failure, SUs wait for beginning of next $T_{cycle}$. The whole working procedure of the proposed scheme is elaborated with the help of flow diagram in Figure 4.

**Algorithm 1** Target channel selection.
1:**procedure**True Prediction(PredProb, RemPkts)2:  **for** SU∈System **do** /* existing SUs */3:   sort(ascend) /* arrange SUs with respect to RemPkts in lowest to highest order*/4:  **end for**5:  **for** Ch $i=1:N_{ch}$ **do**6:   sort(decend) /* arrange all Channels with respect to prob of idleness in highest to lowest order*/7:  **end for**8:  **for** j←1,length(SU) **do**9:   $Ch_{j}\leftarrow SU_{j}$ /*Channel with jth highest prob of idleness is allotted to SU with jth shortest job*/10:   **if** $Ch_{j}$ is sensedidle **then**11:    **transmit** $SU_{j}$ using $Ch_{j}$ /* contention free access as prediction is true*/12:    **remove** $Ch_{j}$ from list of available idle channel13:   **else** *goto Procedure False Prediction*14:   **end if**15:  **end for**16:
**end procedure**
   17:
$SU_{fp}=$ *SUs unsuccessful in channel access due to false prediction*
18:
$SU_{n}=$ *new arriving SUs in the system*
19:
$M_{n}=$ *new arriving SUs in the system*
20:**procedure**False Prediction($M_{n}$, $SU_{n}$, $SU_{fp}$)21:  $contenders\leftarrow SU_{n}+SU_{fp}$22:  $M_{n}\leftarrow listofavailableidlechannels$23:  **begin** contention for channel access/* All contenders contend for channel access in random access fashion*/24:  **if** collision **then**25:   **wait** *until beginning of next $T_{cycle}$*26:  **else**27:   **transmit** *on channel accessed successfully during contention*28:  **end if**29:
**end procedure**



## 4. Simulation and Results

In this section, the performance evaluation of the proposed spectrum handoff scheme and its comparison with NHO and RHO schemes are presented. Simulation parameters used for performance evaluation are presented in Table 3. The basic MAC protocol parameters for the proposed scheme are adopted from IEEE 802.11a [43]. A single slot time (aSlotTime), CR-SIFS (aSIFSTime), CR-RTS and CR-CTS are 9 μs, 16 μs, 24 μs and 24 μs, respectively. Data rate (R) of channel is considered as 54 Mbps, therefore single bit duration (aBitsTime) is 1/R=0.0185 μs. $T_{idle},T_{pred},T_{ss}$ and $T_{cont}$ are calculated using Equations (3)–(6). Total number of channels available to primary network is fixed at 100 and results are obtained by varying the load on primary network (ρ) within a range of [0.0,0.9]. Mean number of contention slots, which is the number of idle channels sensed in each $T_{cycle}$ for contention, depends on the ρ and is calculated as $M=N_{ch}\left(1-\rho \right)$. Mean SU arrival rate $\left(\lambda_{s}\right)$ in each $T_{cycle}$ is varied within a range of [0.5,1,2]. Increasing the $\lambda_{s}$ increases the number of contenders against *M* idle channels in a $T_{cycle}$ and affects the rate of collision among SUs, eventually increasing the EDDT. Similarly, mean number of packets to be transmitted by the newly arriving SU (γ) is considered as [3,5,10]. Increasing the γ requires more time by the SUs to complete transmitting their data packets before departing out of the system. Therefore, for a high value of ρ, most of the channels are occupied by the PUs leaving behind fewer opportunities for the SUs, hence causing SUs to remain in the system for more number of $T_{cycle}$ due to higher number of collisions. Service capacity of the secondary network is measured in terms of average number of packets arriving into the system during a $T_{cycle}$, i.e., $\lambda_{s}\gamma$, and average number of packets departing out of the system after successful transmission served by $\bar{c}$ channel and is denoted by $\mu_{s}=\bar{c}/\gamma$, where $\bar{c}\leq M$ is the average number of channel out of *M* idle channels successfully utilized for data transmission. For a stable system the net arrival rate of packets coming in to the system must be equal or less than the net departure rate of packets out of the system, i.e., $\lambda_{s}\gamma \leq \bar{c}/\gamma$. Congestion in the system increases as $\lambda_{s}\gamma$ increases that can eventually choke the system.

The performance analysis of the proposed scheme and the comparison with NHO and RHO has been conducted in terms of the percentage of collisions among SUs, EDDT, average throughput and cycle time utilization efficiency.

### 4.1. Percentage of Collisions among SUs

**Figure 5:**
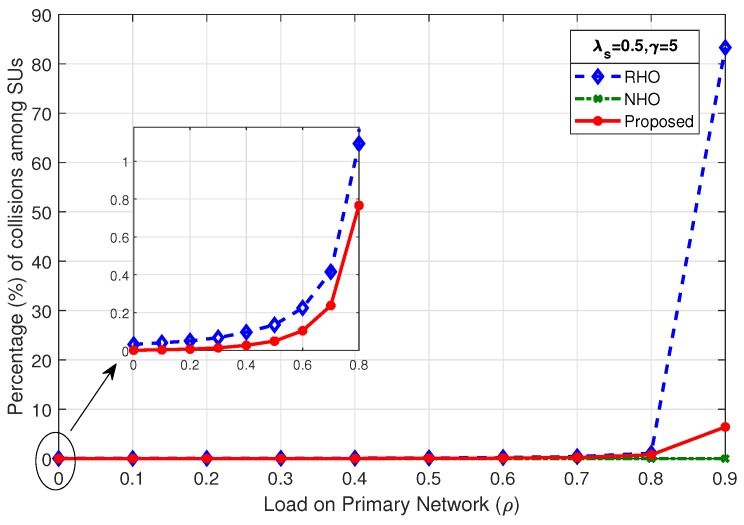
Comparison of percentage of collisions among SUs for $\lambda_{s}=0.5,\gamma =5$.

**Figure 6:**
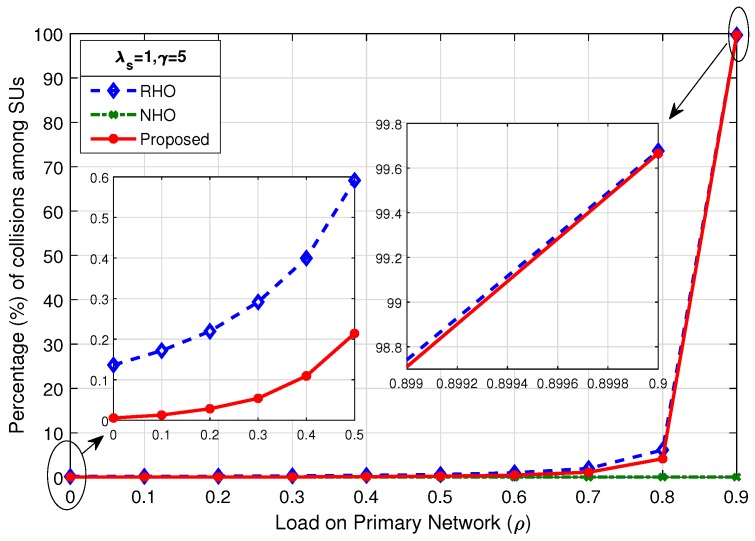
Comparison of percentage of collisions among SUs for $\lambda_{s}=1,\gamma =5$.

**Figure 7:**
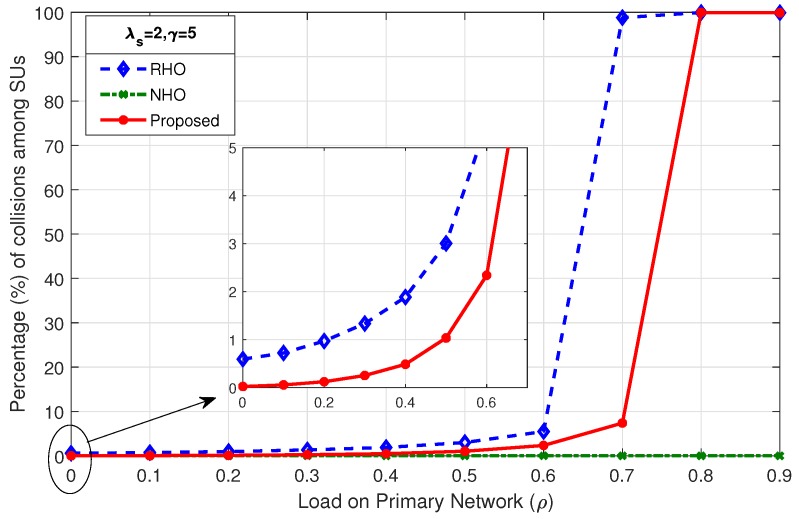
Comparison of percentage of collisions among SUs for $\lambda_{s}=2,\gamma =5$.

The probability of collisions during random channel access when SUs contend for an idle channel is given in Equation (1). The probability of collision increases as the number of contenders increases or the number of available idle channels decreases. The number of idle channel discovered in each $T_{cycle}$ is dependent on the load on primary network (ρ). Figure 5, Figure 6 and Figure 7 show the comparison of percentage of collisions among SUs for RHO, NHO and the proposed scheme. It is observed that the percentage of collisions for NHO remains very low for the entire range of ρ. This is due to the fact that, in NHO, the interrupted SU remains loyal to the channel and instead of switching the channel keeps waiting on the channel until it becomes idle again. Thus, target channel selection is done only until the first successful channel access. The same channel is then used afterwards until SU completes its data transmission. In this case, the collisions are only among the new arriving SUs and existing SUs waiting on the channel. Primarily, collisions occur when SUs contend for a channel in random fashion in an uncoordinated or uncontrolled manner. Therefore, the main comparison for percentage of collisions among SUs was between the proposed scheme and RHO, where SUs attempt to access the channel randomly. The gain in proposed scheme lies in true prediction, where SUs organized according to shortest job first (SJF) principle and access the channel in a coordinated manner. In the proposed scheme, contention-based access is required only when the prediction goes wrong. Figure 5 shows that, as the ρ increases, the percentage of collisions among SUs increases, although it increases very slowly, as depicted in the mini-graph. However, beyond ρ=0.8, the percentage of collisions increases drastically as there are very few opportunities left for SUs. The mini-graph shows better performance for the proposed scheme but the gain achieved in the proposed scheme at high load is much more prominent: at $\rho =0.9,\lambda_{s}=0.5,\gamma =5$, collisions among SUs in proposed scheme is 6.41% compared to 83.28% in RHO scheme. As the SU arrival rate increases to $\lambda_{s}=1$ and $\lambda_{s}=2$, as shown in Figure 6 and Figure 7, respectively, an early rise in the percentage of collisions among SUs is observed. However, the proposed scheme remains better throughout.

### 4.2. Extended Data Delivery Time

**Figure 8:**
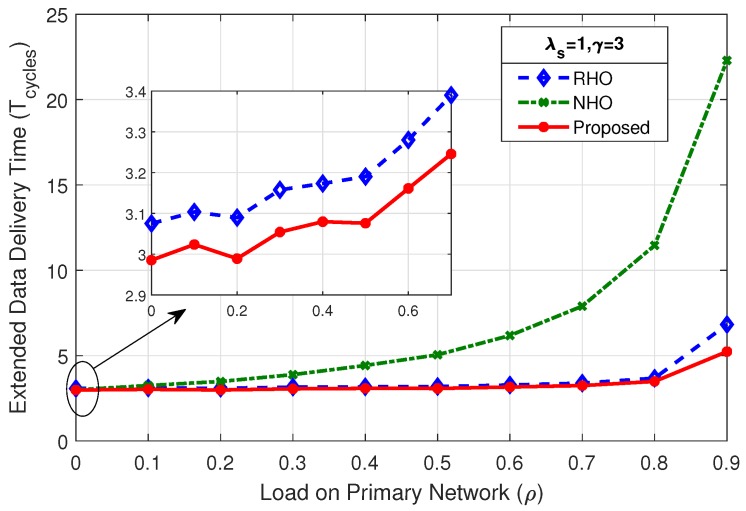
Comparison of mean EDDT for $\lambda_{s}=1,\gamma =3$.

Figure 8 shows the comparison of mean extended data delivery time, which is the time an SU spends in the system in transmitting all of its data packets before departing out of the system. By virtue of reduced collisions among SUs, the proposed scheme shows better performance throughout the considered range of load on primary network (ρ) in terms of EDDT in comparison with RHO and NHO.

The mean EDDT values for RHO and proposed scheme begin with the value close to γ and remain at that level for load on primary up to 80% (ρ=0.8), and sharply rise afterwards. This is due to the fact that reduced opportunities are available for the SUs at higher PU load and result in more collisions and prediction errors. The mean EDDT value for NHO begins with value closer to γ and gradually increases as the load on primary network increases. This is due to the fact that, when ρ increases the particular channel on which the SU stays, under always staying policy, becomes unavailable causing the SU to wait more number of cycles. Further, we discuss in detail the effect of variation of mean number of packets (γ) and SU arrival rate $\left(\lambda_{s}\right)$ on EDDT.

#### 4.2.1. Effect of Mean Number of Packets (γ) on EDDT

**Figure 9:**
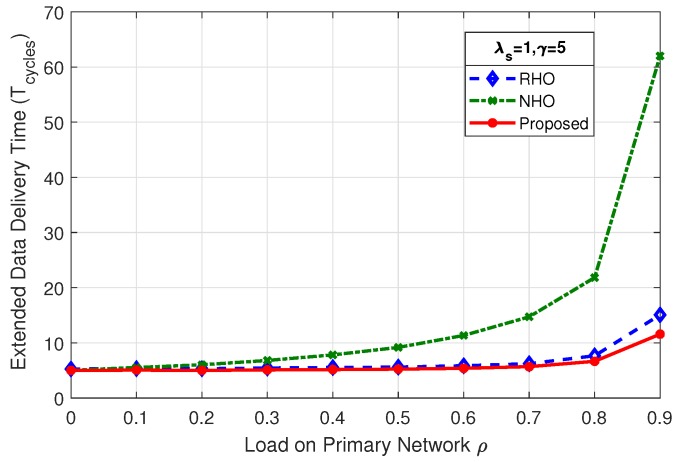
Comparison of mean EDDT for $\lambda_{s}=1,\gamma =5$.

**Figure 10:**
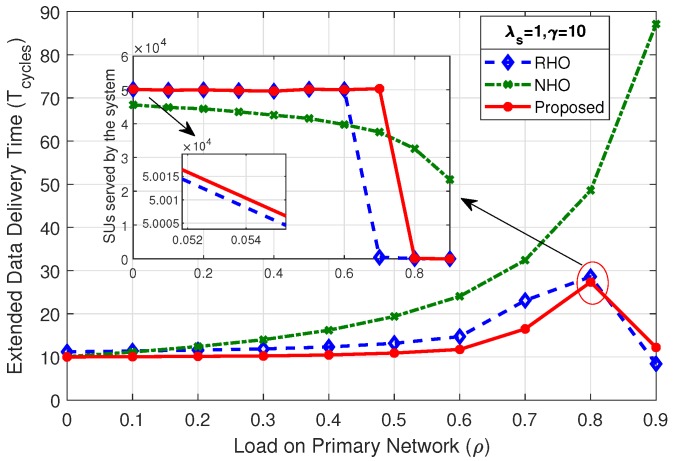
Comparison of mean EDDT for $\lambda_{s}=1,\gamma =10$.

The mean number of packets (γ) transmitted by the arriving SUs has direct impact on the mean EDDT. As the γ increases, SU has to stay in the system for more number of cycles to complete its data transmission. The net rate of packet arrival (packets entering into the system) must be limited in such a way that the system does not destabilize and choke. It demands a higher rate of SU departure out of the system compared to SU arrival rate. As we increase the mean number of packets to be transmitted by SUs to γ=3, γ=5, and γ=10, as in Figure 8, Figure 9 and Figure 10 for the fixed value of $\lambda_{s}$, we observe an increase in the EDDT. The proposed scheme outperforms the other two schemes in comparison; however, at the higher value of γ and high load on the primary network (ρ=0.9), an abrupt behavior is observed for RHO and the proposed scheme. In Figure 10, the dip observed after 80% load on primary network in RHO and the proposed scheme is due to the fact that there are very few idle channels available, resulting in very few SUs being able to complete their data transmission as evident from the mini-graph. The mini graph clearly indicates that the proposed scheme is able to serve a higher number of SUs than that in RHO, yet the abrupt behavior observed is due to the system choking and inability of the proposed simulation model to average out the EDDT, as we average only for the completed jobs. Under identical conditions, NHO is still able to successfully serve some SUs, primarily due to always-staying policy.

At low primary load, the net arrival of packets coming into the systems is less than the packets departing out of the system as SUs are able to discover more idle channels. As the primary load increases the opportunities for the SUs decreases, requiring more time to be spent in the system to complete their transmission. With the system already getting congested, the new arriving SUs continue to pile up in the system, eventually leading to instability and choking of the system. The abrupt behavior in the graph is due to the very same reason and depicts an unstable condition in the system.

#### 4.2.2. Effect of Mean SU Arrival Rate $\left(\lambda_{s}\right)$ on EDDT

**Figure 11:**
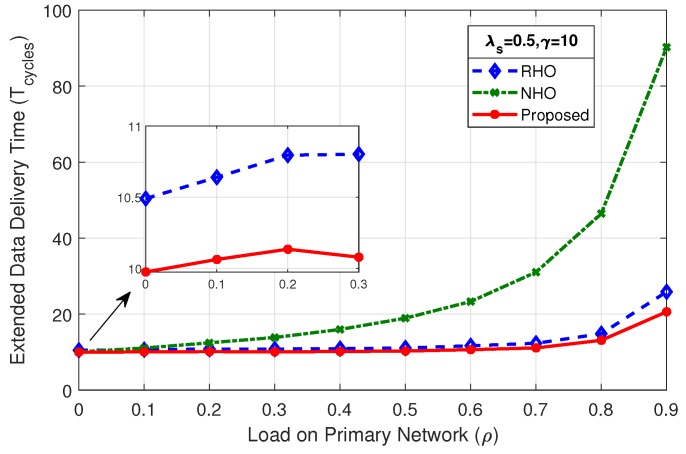
Comparison of mean EDDT for $\lambda_{s}=0.5,\gamma =10$.

As the SU arrival rate $\lambda_{s}$ decreases from 1 to 0.5, keeping the γ fixed, as shown in Figure 10 and Figure 11, respectively, the system remains stable even at higher primary load value, i.e., (ρ=0.9).

Keeping the γ fixed and limiting the SU arrival rate $\lambda_{s}$ reduces the congestion on the CRN, thus allowing higher number of SUs to complete their data transmissions. On the other hand, increasing the $\lambda_{s}$ increases the EDDT because large number of SUs accumulate in the system and in turn increase congestion. Consequently, SUs have to spend more time in order to complete their data transmission for all schemes. Except for the values of load on primary network (ρ) where system chokes due to unavailability of radio channel, the proposed scheme successfully reduces the EDDT.

### 4.3. Average Throughput of the System

Throughput of the system is the amount of data transmitted in a given time period, so, during a $T_{cycle}$, throughput of the system in terms of bps/Hz [18] is:

(16) $$Throughput=\frac{T_{cycle}-T_{overhead}}{T_{cycle}}\log_{2}\left(1+SNR_{su}\right),$$

where $T_{overhead}$ is time consumed in transmitting the control information and $SNR_{su}$ is the signal to noise ratio of the transmitted signal measured at the SU receiver. Therefore, throughput of the system for NHO, RHO and the proposed scheme using Equations (8)–(10), respectively, is:

(17) $$Throughput^{NHO}=\frac{\left(T_{cycle}-T_{overhead}^{NHO}\right)}{T_{cycle}}\log_{2}\left(1+SNR_{su}\right),$$

(18) $$Throughput^{RHO}=\frac{\left(T_{cycle}-T_{overhead}^{RHO}\right)}{T_{cycle}}\log_{2}\left(1+SNR_{su}\right),$$

(19) $$Throughput^{Proposed}=\frac{\left(T_{cycle}-T_{overhead}^{Proposed}\right)}{T_{cycle}}\log_{2}\left(1+SNR_{su}\right).$$

**Figure 12:**
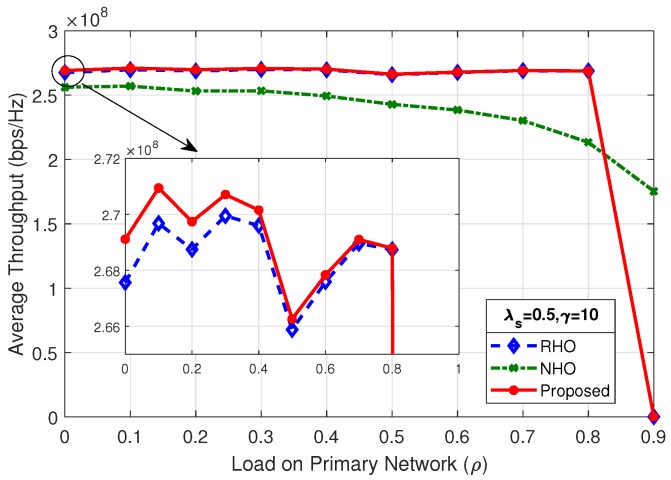
Average throughput of the system for $\lambda_{s}=0.5,\gamma =10$.

**Figure 13:**
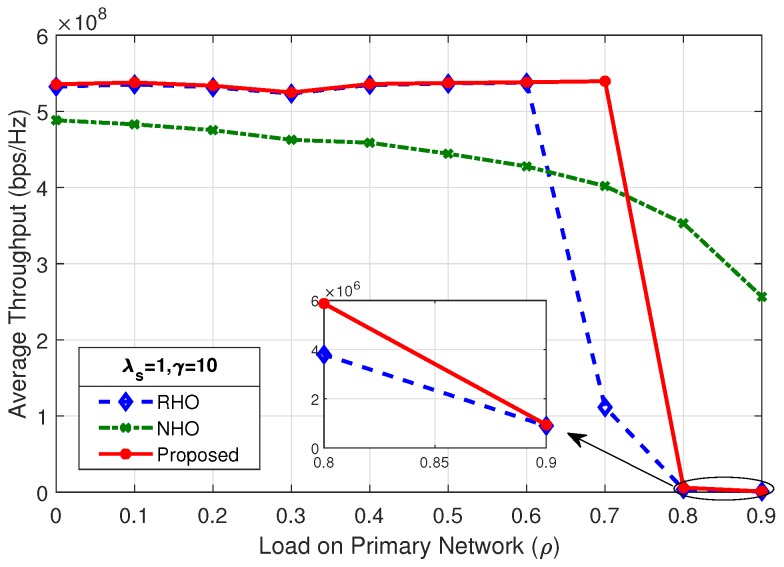
Average throughput of the system for $\lambda_{s}=1,\gamma =10$.

Figure 12 shows the average throughput of the system for $\lambda_{s}=0.5$ and γ=10; the proposed scheme outperform the two other spectrum handoff schemes. The SU arrival rate $\left(\lambda_{s}\right)$ and mean number of packets (γ) has a direct impact on the average throughput of the system. As shown in Figure 13, changing the rate of SU arrival $\left(\lambda_{s}\right)$ from 0.5 to 1, while keeping the mean number of packets (γ) fixed, the maximum average throughput of the system increases by a factor of two, i.e., from 260 Mbps to 535 Mbps. This is due to the fact that doubling the SU arrival rate $\left(\lambda_{s}\right)$ doubles the concurrent SUs in the system. As long as the system is below the choking level, the SUs are able to tap more opportunities, therefore increasing the maximum average throughput of the system.

**Figure 14:**
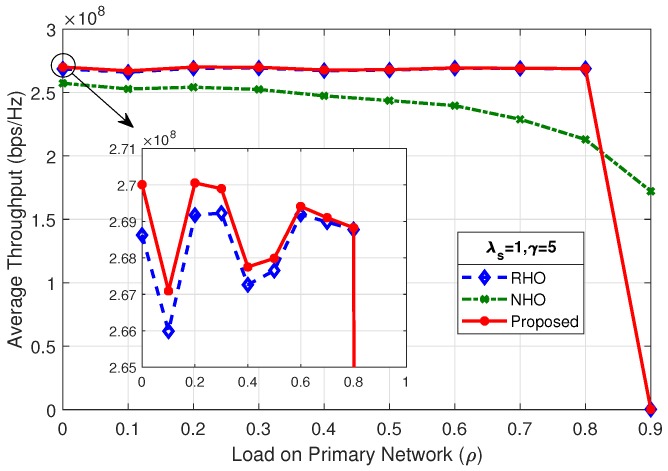
Average throughput of the system for $\lambda_{s}=1,\gamma =5$.

**Figure 15:**
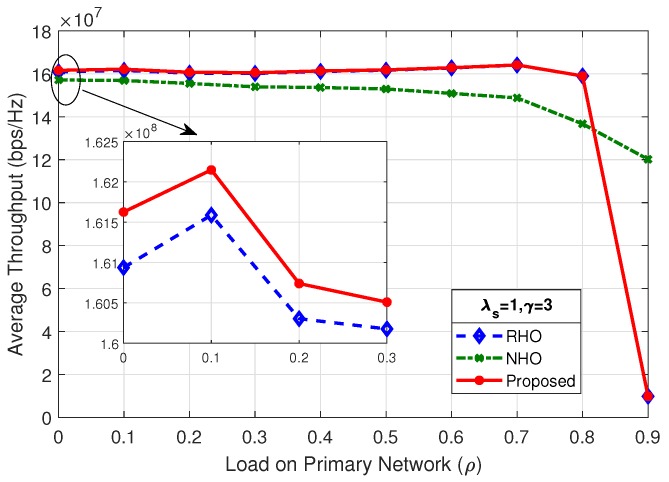
Average throughput of the system for $\lambda_{s}=1,\gamma =3$.

By comparing the results of Figure 13, Figure 14 and Figure 15, it is observed that, as the load on secondary network decreases by reducing the mean number of packets (γ), the system chokes at a higher value of load on the primary network (ρ). Average throughput of the system in Figure 12 and Figure 14 shows identical result as the product of $\lambda_{s}$ and γ are same. It is revealed that the average throughput of the system is dependent on the product of $\lambda_{s}$ and γ.

### 4.4. Cycle Time Utilization Efficiency

SUs utilize only a portion of the cycle time in a given $T_{cycle}$ on the allocated channel. The percentage utilization of $T_{cycle}$ is dependent on the idle, prediction, sensing sharing and contention time, which accounts for total overhead time $\left(T_{overhead}\right)$. Cycle time utilization efficiency $\left(\eta_{T_{cycle}}\right)$ is the ratio of cycle time utilized for transmission $\left(T_{tran}\right)$ and total cycle time $\left(T_{cycle}\right)$. The percentage utilization efficiency for data transmission is given as follows:

(20) $$\eta_{T_{cycle}}=\frac{T_{cycle}-T_{overhead}}{T_{cycle}}*100.$$

Based on Equations (8)–(10) and (20), $\eta_{T_{cycle}}$ for RHO, NHO, and the proposed scheme, respectively, is given as follows:

(21) $$\eta_{T_{cycle}}^{RHO}=\frac{T_{cycle}-T_{overhead}^{RHO}}{T_{cycle}}*100,$$

(22) $$\eta_{T_{cycle}}^{NHO}=\frac{T_{cycle}-T_{overhead}^{NHO}}{T_{cycle}}*100,$$

(23) $$\eta_{T_{cycle}}^{Proposed}=\frac{T_{cycle}-T_{overhead}^{Proposed}}{T_{cycle}}*100.$$

**Figure 16:**
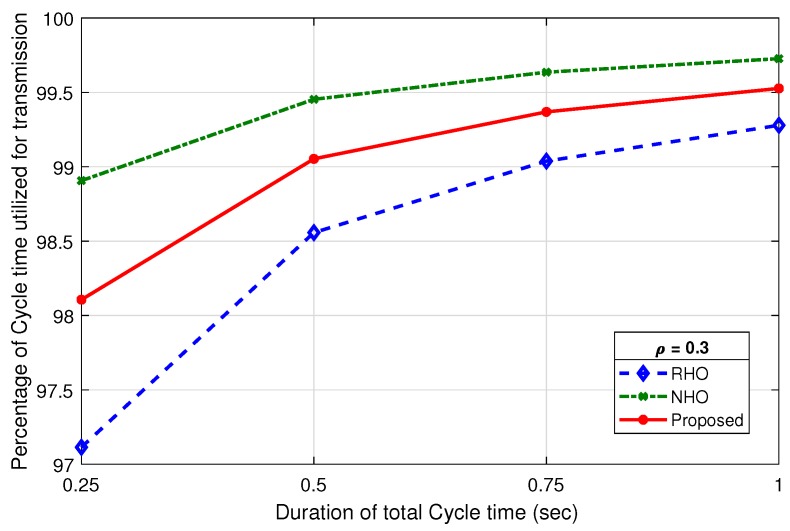
Percentage of cycle time utilization for data transmission against ρ=0.3.

**Figure 17:**
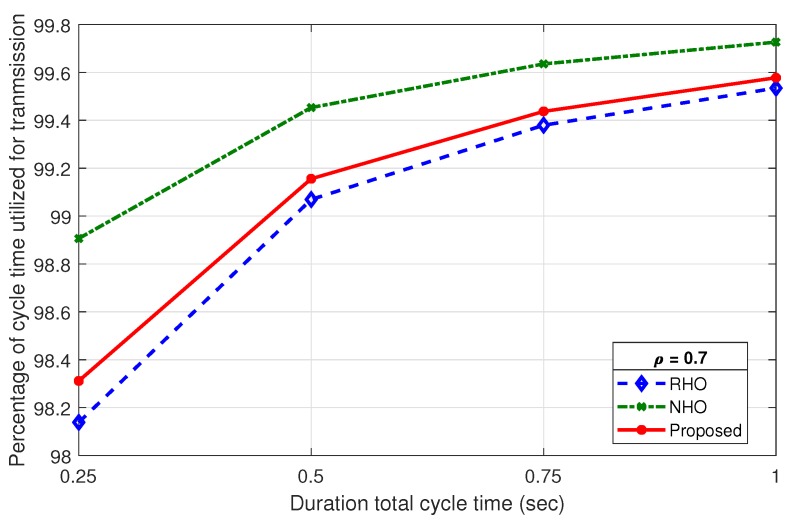
Percentage of cycle time utilization for data transmission against ρ=0.7.

Figure 16 shows a comparison of percentage utilization $\left(\eta_{T_{cycle}}\right)$ of $T_{cycle}$ for RHO, NHO and the proposed scheme at ρ=0.3. The $T_{overhead}^{NHO}$ is fixed for entire range of ρ as it only consists of idle time and sensing and sharing time. However, $T_{overhead}^{RHO}$ and $T_{overhead}^{Proposed}$ are dependent on ρ, as contention period $\left(T_{cont}\right)$ varies with the number of idle channels (M) in a $T_{cycle}$. Due to less overhead, the $\eta_{T_{cycle}}^{NHO}$ is highest for all the values of $T_{cycle}$ duration. The efficiency remains very high throughout for all schemes. Although it is slightly less efficient for proposed scheme than NHO, it is more than compensated in the overall system utilization by virtue of prediction. True prediction not only eliminates contention phase, but also improves channel successful utilization. An identical behavior is observed on increasing ρ, as shown in Figure 17, with a slight improvement for RHO, but still lower than the proposed scheme.

## 5. Conclusions and Future Work

This work has focused on improving the performance of spectrum handoff for the SUs in a cognitive radio ad hoc network (CRAHN) and has developed a proactive partial collision free scheme that is based on imperfect channel state prediction. To the best of our knowledge, this proposed spectrum handoff scheme is the first attempt to include a prediction model that takes into consideration the imperfect channel state for reducing the collisions among the SUs, minimizing the extended data delivery time (EDDT) and improving throughput of the SUs in the network. Performance evaluation indicated that the proposed scheme reduces the EDDT compared to that in the random access spectrum handoff and non-handoff schemes by providing collision free access to a certain number of the SUs. The analysis has also revealed that by improving the channel state prediction, increased contention free channel access can be granted, which reduces the collisions on the remaining idle channels for the rest of the SUs. This work can further be extended in several directions that include use of artificial neural network (ANN) or hidden Markov model (HMM) based channel state prediction techniques, incorporation of imperfect sensing in the model (the current scheme has assumed perfect sensing for simplicity), consideration of mobility of SUs and adoption of a priority-oriented class-of-service approach for various types of traffic that can afford quality of service (QoS) guarantees to the time-constrained applications in a CRAHN.

## Figures and Tables

**Table 1:** Comparison of related work and proposed work in terms of imperfect spectrum prediction based spectrum handoff with collision reduction in CRAHN.

	Ad hoc Network	Improved Frame Format	Channel Access Re-attempt within Same Time Cycle	Imperfect Prediction	Consideration of Prediction for Handoff Management	Channel Selection Mechanism	Throughput	EDDT	Collision Among SUs	Cycle Time Utilization Efficiency
Aggarwal et al. [21] (2019)	✗	✗	✗	✓	✓	✓	✗	✓	✓	✗
Tayel et al. [11] (2018)	✗	✗	✗	✗	✗	✓	✓	✓	✗	✗
Nguyen et al. [16] (2018)	✗	✗	✗	✓	✗	✗	✓	✗	✗	✗
Mir et al. [14] (2017)	✗	✗	✗	✗	✓	✓	✓	✓	✗	✗
Shil et al. [20] (2017)	✗	✗	✗	✓	✓	✓	✓	✓	✗	✗
Usman et al. [15] (2015)	✗	✗	✗	✗	✓	✓	✓	✓	✗	✗
Yang et al. [18] (2015)	✓	✓	✗	✓	✗	✗	✓	✗	✓	✗
Wang et al. [19] (2015)	✗	✗	✗	✓	✗	✗	✓	✗	✗	✗
Tumuluru et al. [17] (2012)	✗	✗	✗	✓	✗	✓	✓	✗	✗	✗
Song et al. [25] (2012)	✓	✗	✗	✓	✓	✓	✗	✓	✓	✗
Wang et al. [12] (2012)	✗	✗	✗	✗	✓	✓	✓	✓	✓	✗
Song et al. [13] (2010)	✓	✗	✗	✓	✓	✓	✗	✓	✓	✗
Proposed	✓	✓	✓	✓	✓	✓	✓	✓	✓	✓

**Table 2:** True channel state and prediction probabilities.

True Channel State (Sensing)	Prediction	Probability
*Idle*	*Idle*	$P\left(H_{0}\right)\left(1-P_{p}^{e}\right)$
*Idle*	*Busy*	$P\left(H_{0}\right)\left(P_{p}^{e}\right)$
*Busy*	*Idle*	$P\left(H_{1}\right)\left(P_{p}^{e}\right)$
*Busy*	*Busy*	$P\left(H_{1}\right)\left(1-P_{p}^{e}\right)$

**Table 3:** Simulation parameters.

Simulation Parameters	
Simulation Time	100,000 Cycles
No. of primary channels ($N_{ch}$)	100
Load on primary network (ρ)	[0.1–0.9]
Mean SU arrival rate in each $T_{cycle}$ ($\lambda_{s}$)	[0.5, 1, 2]
Mean contention slots (*M*)	$N_{ch}*\left(1-\rho \right)$
Mean number of packets to transmit by SU (γ)	[3, 5, 10]
Probability threshold ($P_{Th}$)	0.5
Data rate of the channel (*R*)	54 Mbps
Single slot time (aSlotTime)	9 μs
CR-SIFS time (aSIFSTime)	16 μs
CR-RTS	24 μs
CR-CTS	24 μs
Single bit time (aBitsTime)	0.0185 μs
Idle phase time ($T_{idle}$)	34 μs
Prediction phase time ($T_{pred}$)	0.277777 μs
Sensing and Sharing phase time ($T_{ss}$)	$N_{ch}*27$ μs
Contention phase time ($T_{cont}$)	M∗64 μs

## Abbreviations

The following abbreviations are used in this manuscript:

ANN	Artificial Neural Network
CCC	Common Control Channel
CRAHN	Cognitive Radio Ad Hoc Network
CRN	Cognitive Radio Network
CTS	Clear to Send
DSA	Dynamic Spectrum Access
EDDT	Extended Data Delivery Time
FCC	Federal Communication Commissions
FCFS	First Come First Serve
HMM	Hidden Markov Model
IoT	Internet of Things
NHO	Non-Handoff
PRP	Preemptive Resume Priority
PUs	Primary Users
RHO	Random Handoff
RTS	Request to Send
SIFS	Short Inter Frame Space
SJF	Shortest Job First
SUs	Secondary Users
